# Di-μ-nitrito-κ^3^ *O*:*O*,*O*′;κ^3^ *O*,*O*′:*O*-bis­{[2,6-bis­(pyrazol-1-yl-κ*N* ^2^)pyridine-κ*N*](nitrito-κ^2^ *O*,*O*′)cadmium(II)}

**DOI:** 10.1107/S1600536809039841

**Published:** 2009-10-07

**Authors:** Ting Ting Sun, Lin Meng, Jing Min Shi

**Affiliations:** a Department of Chemistry, Shandong Normal University, Jinan 250014, People’s Republic of China

## Abstract

In the title centrosymmetric binuclear complex, [Cd_2_(NO_2_)_4_(C_11_H_9_N_5_)_2_], the unique Cd^II^ ion is in a distorted dodeca­hedral CdN_3_O_5_ coordination environment. The two inversion-related Cd^II^ ions are separated by 3.9920 (6) Å and are bridged by two O atoms from two nitrite ligands. There are two types of π–π stacking inter­actions involving symmetry-related pyrazole rings, with centroid–centroid distances of 3.445 (2) and 3.431 (2) Å.

## Related literature

For related structures, see: Yang & Sun (2008 ▶); Bessel *et al.* (1993 ▶).
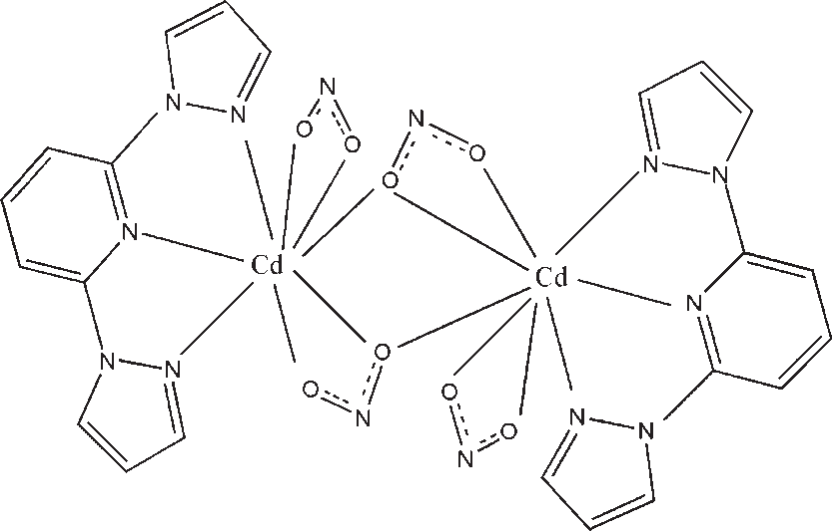


## Experimental

### 

#### Crystal data

[Cd_2_(NO_2_)_4_(C_11_H_9_N_5_)_2_]
*M* *_r_* = 831.30
Triclinic, 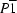

*a* = 7.7618 (13) Å
*b* = 9.5522 (16) Å
*c* = 10.9665 (19) Å
α = 110.285 (2)°
β = 90.616 (2)°
γ = 112.155 (2)°
*V* = 696.9 (2) Å^3^
*Z* = 1
Mo *K*α radiation
μ = 1.60 mm^−1^
*T* = 298 K
0.32 × 0.21 × 0.10 mm

#### Data collection

Bruker SMART APEX CCD diffractometer
Absorption correction: multi-scan (*SADABS*; Sheldrick, 1996 ▶) *T*_min_ = 0.628, *T*_max_ = 0.856
3815 measured reflections
2666 independent reflections
2468 reflections with *I* > 2σ(*I*)
*R*_int_ = 0.017

#### Refinement

*R*[*F* ^2^ > 2σ(*F* ^2^)] = 0.029
*wR*(*F* ^2^) = 0.076
*S* = 1.09
2666 reflections
208 parameters
H-atom parameters constrained
Δρ_max_ = 0.56 e Å^−3^
Δρ_min_ = −0.53 e Å^−3^

### 

Data collection: *SMART* (Bruker, 1997 ▶); cell refinement: *SAINT* (Bruker, 1997 ▶); data reduction: *SAINT*; program(s) used to solve structure: *SHELXTL* (Sheldrick, 2008 ▶); program(s) used to refine structure: *SHELXTL*; molecular graphics: *SHELXTL*; software used to prepare material for publication: *SHELXTL*.

## Supplementary Material

Crystal structure: contains datablocks I, global. DOI: 10.1107/S1600536809039841/lh2917sup1.cif

Structure factors: contains datablocks I. DOI: 10.1107/S1600536809039841/lh2917Isup2.hkl

Additional supplementary materials:  crystallographic information; 3D view; checkCIF report

## Figures and Tables

**Fig. 1.**
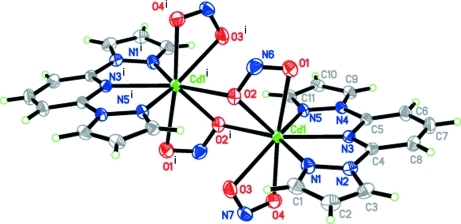
The molecular structure of title compound with the atom-numbering scheme. Displacement ellipsoids are drawn at the 30% probability level. Symmetry code: (i) 1 - x, 2 - y, 2 - z

**Fig. 2.**
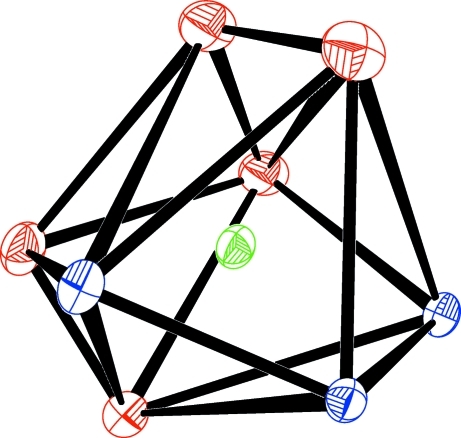
The coordination environment of CdII

## supplementary crystallographic information

### Comment

2,6-Dipyrazol-1-ylpyridine is expected be a useful tridentate ligand, but complexes with it as ligand to our knowledge are somewhat rare (e.g. Yang & Sun, 2008; Bessel et al.,  1993). Our interest in complexes with 2,6-dipyrazol-1-ylpyridine as a ligand has motivated us to prepare the title complex, (I), and herein we report its crystal structure.

Fig. 1 shows the molecular structure of the title complex. Each Cd^II^ ion is coordinated by five O atoms and three N atoms in a distorted dodecahedral coordination environment (see Fig. 2). It is rare for Cd^II^ to assume this coordination mode. Fig. 1 also shows that two nitrite anions function as bridging ligands, linking two inversion related Cd^II^ ions with a separation of 3.9920 (6) Å leading to a binuclear Cd^II^ complex. In the crystal there are the strong π–π stacking interactions involving the symmetry related triazole rings, with the relevant distances being Cg1···Cg2^i^ = 3.445 (2) Å, Cg2···Cg2^ii^ =  3.431 (2) Å, Cg1···Cg2^i^_perp_ = 3.299 Å and Cg2···Cg2^ii^_perp_ = 3.274 Å (symmetric codes: (i) -1+x, y, z; (ii) 1-x, 1-y, 2-z; Cg1 and Cg2 are the centroids of C1-C3/N1/N2; C9-C11/N4/N5 triazole rings, respectively; Cgi···Cgj_perp_ is the perpendicular distance from Cgi ring to Cgj ring).

### Experimental

10 ml dichloromethane solution of 2,6-Dipyrazol-1-ylpyridine (0.0692 g, 0.328 mmol) was added into 10 ml methanol solution containing Cd(ClO_4_).6H_2_O (0.0740 g, 0.176 mmol) and sodium nitrite (0.0138 g, 0.200 mmol) and the mixed soluton was stirred for a few minutes. The colorless single crystals were obtained after the filtrate had been allowed to stand at room temperature for about one month.

### Refinement

All H atoms were placed in calculated positions and refined as riding with C—H = 0.93 Å, *U*_iso_ = 1.2*U*_eq_(C).

### Crystal data

**Table:**

[Cd_2_(NO_2_)_4_(C_11_H_9_N_5_)_2_]	*Z* = 1
*M**_r_* = 831.30	*F*(000) = 408
Triclinic, *P*1	*D*_x_ = 1.981 Mg m^−^^3^
Hall symbol: -P 1	Mo *K*α radiation, λ = 0.71073 Å
*a* = 7.7618 (13) Å	Cell parameters from 2504 reflections
*b* = 9.5522 (16) Å	θ = 2.5–27.8°
*c* = 10.9665 (19) Å	µ = 1.60 mm^−^^1^
α = 110.285 (2)°	*T* = 298 K
β = 90.616 (2)°	Block, colorless
γ = 112.155 (2)°	0.32 × 0.21 × 0.10 mm
*V* = 696.9 (2) Å^3^	

### Data collection

**Table:**

Bruker SMART APEX CCD diffractometer	2666 independent reflections
Radiation source: fine-focus sealed tube	2468 reflections with *I* > 2σ(*I*)
graphite	*R*_int_ = 0.017
φ and ω scans	θ_max_ = 26.0°, θ_min_ = 2.0°
Absorption correction: multi-scan (*SADABS*; Sheldrick, 1996)	*h* = −9→9
*T*_min_ = 0.628, *T*_max_ = 0.856	*k* = −11→11
3815 measured reflections	*l* = −7→13

### Refinement

**Table:**

Refinement on *F*^2^	Primary atom site location: structure-invariant direct methods
Least-squares matrix: full	Secondary atom site location: difference Fourier map
*R*[*F*^2^ > 2σ(*F*^2^)] = 0.029	Hydrogen site location: inferred from neighbouring sites
*wR*(*F*^2^) = 0.076	H-atom parameters constrained
*S* = 1.09	*w* = 1/[σ^2^(*F*_o_^2^) + (0.044*P*)^2^] where *P* = (*F*_o_^2^ + 2*F*_c_^2^)/3
2666 reflections	(Δ/σ)_max_ = 0.002
208 parameters	Δρ_max_ = 0.56 e Å^−^^3^
0 restraints	Δρ_min_ = −0.53 e Å^−^^3^

### Special details

**Table:**

Geometry. All e.s.d.'s (except the e.s.d. in the dihedral angle between two l.s. planes) are estimated using the full covariance matrix. The cell e.s.d.'s are taken into account individually in the estimation of e.s.d.'s in distances, angles and torsion angles; correlations between e.s.d.'s in cell parameters are only used when they are defined by crystal symmetry. An approximate (isotropic) treatment of cell e.s.d.'s is used for estimating e.s.d.'s involving l.s. planes.
Refinement. Refinement of *F*^2^ against ALL reflections. The weighted *R*-factor *wR* and goodness of fit *S* are based on *F*^2^, conventional *R*-factors *R* are based on *F*, with *F* set to zero for negative *F*^2^. The threshold expression of *F*^2^ > σ(*F*^2^) is used only for calculating *R*-factors(gt) *etc*. and is not relevant to the choice of reflections for refinement. *R*-factors based on *F*^2^ are statistically about twice as large as those based on *F*, and *R*- factors based on ALL data will be even larger.

### Fractional atomic coordinates and isotropic or equivalent isotropic displacement parameters (Å^2^)

**Table:**

	*x*	*y*	*z*	*U*_iso_*/*U*_eq_	
C1	−0.0700 (5)	0.7853 (5)	0.7004 (4)	0.0445 (8)	
H1	−0.0364	0.8974	0.7345	0.053*	
C2	−0.2364 (5)	0.6713 (5)	0.6144 (4)	0.0454 (8)	
H2	−0.3315	0.6920	0.5817	0.055*	
C3	−0.2301 (4)	0.5239 (5)	0.5885 (3)	0.0431 (8)	
H3	−0.3207	0.4227	0.5338	0.052*	
C4	0.0015 (4)	0.4386 (4)	0.6698 (3)	0.0296 (6)	
C5	0.2520 (4)	0.4068 (3)	0.7464 (3)	0.0298 (6)	
C6	0.1560 (4)	0.2398 (4)	0.7011 (4)	0.0405 (7)	
H6	0.2125	0.1736	0.7117	0.049*	
C7	−0.0287 (5)	0.1747 (4)	0.6390 (3)	0.0449 (8)	
H7	−0.0984	0.0623	0.6074	0.054*	
C8	−0.1103 (5)	0.2735 (4)	0.6234 (3)	0.0410 (8)	
H8	−0.2350	0.2311	0.5835	0.049*	
C9	0.5554 (4)	0.4211 (4)	0.8411 (3)	0.0357 (7)	
H9	0.5291	0.3105	0.8142	0.043*	
C10	0.7180 (4)	0.5479 (4)	0.9151 (3)	0.0373 (7)	
H10	0.8247	0.5419	0.9481	0.045*	
C11	0.6898 (4)	0.6888 (4)	0.9306 (3)	0.0364 (7)	
H11	0.7781	0.7945	0.9778	0.044*	
Cd1	0.35091 (3)	0.79984 (2)	0.84217 (2)	0.03281 (10)	
N1	0.0340 (4)	0.7146 (3)	0.7277 (3)	0.0374 (6)	
N2	−0.0654 (3)	0.5526 (3)	0.6580 (3)	0.0340 (6)	
N3	0.1788 (3)	0.5057 (3)	0.7307 (2)	0.0290 (5)	
N4	0.4388 (3)	0.4869 (3)	0.8142 (2)	0.0291 (5)	
N5	0.5215 (3)	0.6530 (3)	0.8697 (2)	0.0323 (5)	
N6	0.2056 (4)	0.8778 (4)	1.0874 (3)	0.0506 (7)	
N7	0.4464 (4)	0.9665 (4)	0.6637 (3)	0.0485 (7)	
O1	0.1706 (4)	0.7339 (3)	1.0265 (3)	0.0568 (7)	
O2	0.3107 (4)	0.9700 (3)	1.0329 (3)	0.0542 (7)	
O3	0.3877 (4)	1.0225 (3)	0.7663 (3)	0.0506 (6)	
O4	0.4610 (4)	0.8348 (3)	0.6500 (3)	0.0491 (6)	

### Atomic displacement parameters (Å^2^)

**Table:**

	*U*^11^	*U*^22^	*U*^33^	*U*^12^	*U*^13^	*U*^23^
C1	0.0479 (19)	0.048 (2)	0.054 (2)	0.0301 (17)	0.0146 (16)	0.0260 (18)
C2	0.0422 (18)	0.062 (2)	0.048 (2)	0.0337 (17)	0.0107 (15)	0.0256 (18)
C3	0.0323 (16)	0.056 (2)	0.0377 (18)	0.0187 (16)	0.0004 (14)	0.0135 (16)
C4	0.0314 (14)	0.0328 (15)	0.0262 (14)	0.0157 (13)	0.0086 (12)	0.0100 (12)
C5	0.0325 (14)	0.0285 (14)	0.0298 (14)	0.0139 (12)	0.0074 (12)	0.0110 (12)
C6	0.0434 (18)	0.0305 (15)	0.0486 (19)	0.0162 (14)	0.0023 (15)	0.0147 (14)
C7	0.0458 (19)	0.0280 (16)	0.051 (2)	0.0087 (14)	−0.0021 (16)	0.0111 (15)
C8	0.0353 (16)	0.0388 (18)	0.0395 (18)	0.0087 (14)	−0.0009 (14)	0.0112 (15)
C9	0.0379 (16)	0.0376 (16)	0.0449 (18)	0.0231 (14)	0.0152 (14)	0.0219 (15)
C10	0.0312 (15)	0.0472 (19)	0.0455 (18)	0.0210 (14)	0.0100 (14)	0.0256 (16)
C11	0.0314 (15)	0.0367 (16)	0.0412 (17)	0.0109 (13)	0.0041 (13)	0.0182 (14)
Cd1	0.03791 (15)	0.02645 (14)	0.03535 (15)	0.01474 (11)	0.00396 (10)	0.01137 (10)
N1	0.0360 (14)	0.0340 (14)	0.0457 (16)	0.0174 (12)	0.0059 (12)	0.0158 (12)
N2	0.0312 (13)	0.0396 (14)	0.0345 (14)	0.0175 (12)	0.0061 (11)	0.0144 (12)
N3	0.0303 (12)	0.0283 (12)	0.0305 (13)	0.0138 (10)	0.0043 (10)	0.0115 (10)
N4	0.0302 (12)	0.0271 (12)	0.0338 (13)	0.0136 (10)	0.0055 (10)	0.0137 (11)
N5	0.0324 (13)	0.0262 (12)	0.0396 (14)	0.0120 (11)	0.0054 (11)	0.0138 (11)
N6	0.0513 (18)	0.0524 (19)	0.0435 (17)	0.0200 (15)	0.0137 (14)	0.0140 (15)
N7	0.0554 (18)	0.0436 (17)	0.0508 (18)	0.0175 (15)	0.0031 (15)	0.0257 (15)
O1	0.0566 (16)	0.0422 (15)	0.0591 (17)	0.0080 (13)	0.0077 (13)	0.0181 (14)
O2	0.0708 (17)	0.0338 (13)	0.0464 (14)	0.0131 (12)	0.0090 (13)	0.0108 (11)
O3	0.0584 (15)	0.0340 (12)	0.0602 (17)	0.0202 (12)	0.0044 (13)	0.0171 (12)
O4	0.0610 (16)	0.0464 (14)	0.0462 (14)	0.0291 (13)	0.0099 (12)	0.0166 (12)

### Geometric parameters (Å, °)

**Table:**

C1—N1	1.320 (4)	C9—C10	1.364 (4)
C1—C2	1.395 (5)	C9—H9	0.9300
C1—H1	0.9300	C10—C11	1.397 (5)
C2—C3	1.357 (5)	C10—H10	0.9300
C2—H2	0.9300	C11—N5	1.325 (4)
C3—N2	1.364 (4)	C11—H11	0.9300
C3—H3	0.9300	Cd1—O2	2.270 (3)
C4—N3	1.330 (4)	Cd1—N5	2.345 (2)
C4—C8	1.379 (4)	Cd1—O4	2.365 (3)
C4—N2	1.413 (4)	Cd1—N3	2.434 (2)
C5—N3	1.328 (4)	Cd1—N1	2.450 (3)
C5—C6	1.376 (4)	Cd1—O3	2.464 (2)
C5—N4	1.409 (4)	Cd1—O1	2.592 (3)
C6—C7	1.385 (4)	N1—N2	1.357 (4)
C6—H6	0.9300	N4—N5	1.361 (3)
C7—C8	1.371 (5)	N6—O1	1.220 (4)
C7—H7	0.9300	N6—O2	1.277 (4)
C8—H8	0.9300	N7—O3	1.240 (4)
C9—N4	1.361 (4)	N7—O4	1.264 (4)
			
N1—C1—C2	111.8 (3)	O4—Cd1—N3	93.58 (9)
N1—C1—H1	124.1	O2—Cd1—N1	94.34 (10)
C2—C1—H1	124.1	N5—Cd1—N1	132.58 (9)
C3—C2—C1	105.3 (3)	O4—Cd1—N1	86.53 (9)
C3—C2—H2	127.3	N3—Cd1—N1	65.49 (8)
C1—C2—H2	127.3	O2—Cd1—O3	83.63 (9)
C2—C3—N2	106.8 (3)	N5—Cd1—O3	139.36 (9)
C2—C3—H3	126.6	O4—Cd1—O3	51.23 (9)
N2—C3—H3	126.6	N3—Cd1—O3	130.34 (8)
N3—C4—C8	124.0 (3)	N1—Cd1—O3	77.14 (8)
N3—C4—N2	113.9 (3)	O2—Cd1—O1	50.02 (9)
C8—C4—N2	122.2 (3)	N5—Cd1—O1	87.76 (9)
N3—C5—C6	123.6 (3)	O4—Cd1—O1	169.63 (9)
N3—C5—N4	114.4 (2)	N3—Cd1—O1	80.15 (8)
C6—C5—N4	122.0 (3)	N1—Cd1—O1	83.36 (9)
C5—C6—C7	117.0 (3)	O3—Cd1—O1	127.86 (9)
C5—C6—H6	121.5	C1—N1—N2	104.8 (3)
C7—C6—H6	121.5	C1—N1—Cd1	137.4 (2)
C8—C7—C6	121.0 (3)	N2—N1—Cd1	117.31 (18)
C8—C7—H7	119.5	N1—N2—C3	111.2 (3)
C6—C7—H7	119.5	N1—N2—C4	120.0 (2)
C7—C8—C4	116.8 (3)	C3—N2—C4	128.7 (3)
C7—C8—H8	121.6	C5—N3—C4	117.6 (3)
C4—C8—H8	121.6	C5—N3—Cd1	119.45 (19)
N4—C9—C10	107.1 (3)	C4—N3—Cd1	122.21 (19)
N4—C9—H9	126.4	C9—N4—N5	111.0 (2)
C10—C9—H9	126.4	C9—N4—C5	128.9 (3)
C9—C10—C11	105.3 (3)	N5—N4—C5	120.0 (2)
C9—C10—H10	127.4	C11—N5—N4	105.1 (2)
C11—C10—H10	127.4	C11—N5—Cd1	136.3 (2)
N5—C11—C10	111.5 (3)	N4—N5—Cd1	118.55 (17)
N5—C11—H11	124.2	O1—N6—O2	112.5 (3)
C10—C11—H11	124.2	O3—N7—O4	113.2 (3)
O2—Cd1—N5	114.66 (9)	N6—O1—Cd1	91.5 (2)
O2—Cd1—O4	133.53 (9)	N6—O2—Cd1	105.9 (2)
N5—Cd1—O4	97.45 (9)	N7—O3—Cd1	95.73 (19)
O2—Cd1—N3	128.85 (8)	N7—O4—Cd1	99.9 (2)
N5—Cd1—N3	67.11 (8)		
			
N1—C1—C2—C3	−0.3 (4)	O1—Cd1—N3—C4	83.5 (2)
C1—C2—C3—N2	0.2 (4)	C10—C9—N4—N5	−0.4 (3)
N3—C5—C6—C7	−1.7 (5)	C10—C9—N4—C5	−176.8 (3)
N4—C5—C6—C7	178.2 (3)	N3—C5—N4—C9	−176.9 (3)
C5—C6—C7—C8	0.3 (5)	C6—C5—N4—C9	3.2 (5)
C6—C7—C8—C4	1.3 (5)	N3—C5—N4—N5	7.0 (4)
N3—C4—C8—C7	−1.8 (5)	C6—C5—N4—N5	−172.9 (3)
N2—C4—C8—C7	179.1 (3)	C10—C11—N5—N4	0.2 (4)
N4—C9—C10—C11	0.5 (4)	C10—C11—N5—Cd1	179.2 (2)
C9—C10—C11—N5	−0.5 (4)	C9—N4—N5—C11	0.1 (3)
C2—C1—N1—N2	0.3 (4)	C5—N4—N5—C11	176.8 (3)
C2—C1—N1—Cd1	171.8 (2)	C9—N4—N5—Cd1	−179.10 (18)
O2—Cd1—N1—C1	55.0 (4)	C5—N4—N5—Cd1	−2.4 (3)
N5—Cd1—N1—C1	−175.4 (3)	O2—Cd1—N5—C11	−56.5 (3)
O4—Cd1—N1—C1	−78.5 (3)	O4—Cd1—N5—C11	89.0 (3)
N3—Cd1—N1—C1	−174.0 (4)	N3—Cd1—N5—C11	179.8 (3)
O3—Cd1—N1—C1	−27.5 (3)	N1—Cd1—N5—C11	−178.9 (3)
O1—Cd1—N1—C1	103.8 (3)	O3—Cd1—N5—C11	53.8 (4)
O2—Cd1—N1—N2	−134.3 (2)	O1—Cd1—N5—C11	−100.0 (3)
N5—Cd1—N1—N2	−4.7 (3)	O2—Cd1—N5—N4	122.4 (2)
O4—Cd1—N1—N2	92.3 (2)	O4—Cd1—N5—N4	−92.1 (2)
N3—Cd1—N1—N2	−3.3 (2)	N3—Cd1—N5—N4	−1.34 (19)
O3—Cd1—N1—N2	143.2 (2)	N1—Cd1—N5—N4	0.1 (3)
O1—Cd1—N1—N2	−85.4 (2)	O3—Cd1—N5—N4	−127.3 (2)
C1—N1—N2—C3	−0.1 (4)	O1—Cd1—N5—N4	78.9 (2)
Cd1—N1—N2—C3	−173.6 (2)	O2—N6—O1—Cd1	−3.4 (3)
C1—N1—N2—C4	−176.7 (3)	O2—Cd1—O1—N6	2.3 (2)
Cd1—N1—N2—C4	9.8 (3)	N5—Cd1—O1—N6	127.6 (2)
C2—C3—N2—N1	−0.1 (4)	O4—Cd1—O1—N6	−112.0 (5)
C2—C3—N2—C4	176.1 (3)	N3—Cd1—O1—N6	−165.3 (2)
N3—C4—N2—N1	−12.6 (4)	N1—Cd1—O1—N6	−99.1 (2)
C8—C4—N2—N1	166.5 (3)	O3—Cd1—O1—N6	−31.1 (3)
N3—C4—N2—C3	171.5 (3)	O1—N6—O2—Cd1	4.0 (3)
C8—C4—N2—C3	−9.4 (5)	N5—Cd1—O2—N6	−66.1 (2)
C6—C5—N3—C4	1.3 (5)	O4—Cd1—O2—N6	164.6 (2)
N4—C5—N3—C4	−178.6 (2)	N3—Cd1—O2—N6	13.5 (3)
C6—C5—N3—Cd1	171.7 (3)	N1—Cd1—O2—N6	75.3 (2)
N4—C5—N3—Cd1	−8.2 (3)	O3—Cd1—O2—N6	151.8 (2)
C8—C4—N3—C5	0.6 (4)	O1—Cd1—O2—N6	−2.3 (2)
N2—C4—N3—C5	179.7 (3)	O4—N7—O3—Cd1	−1.1 (3)
C8—C4—N3—Cd1	−169.6 (2)	O2—Cd1—O3—N7	168.8 (2)
N2—C4—N3—Cd1	9.5 (3)	N5—Cd1—O3—N7	47.8 (3)
O2—Cd1—N3—C5	−98.7 (2)	O4—Cd1—O3—N7	0.71 (19)
N5—Cd1—N3—C5	5.3 (2)	N3—Cd1—O3—N7	−54.0 (2)
O4—Cd1—N3—C5	101.9 (2)	N1—Cd1—O3—N7	−95.2 (2)
N1—Cd1—N3—C5	−173.6 (2)	O1—Cd1—O3—N7	−166.11 (18)
O3—Cd1—N3—C5	141.5 (2)	O3—N7—O4—Cd1	1.2 (3)
O1—Cd1—N3—C5	−86.4 (2)	O2—Cd1—O4—N7	−17.1 (3)
O2—Cd1—N3—C4	71.3 (2)	N5—Cd1—O4—N7	−151.9 (2)
N5—Cd1—N3—C4	175.2 (2)	N3—Cd1—O4—N7	140.7 (2)
O4—Cd1—N3—C4	−88.2 (2)	N1—Cd1—O4—N7	75.6 (2)
N1—Cd1—N3—C4	−3.6 (2)	O3—Cd1—O4—N7	−0.70 (19)
O3—Cd1—N3—C4	−48.5 (3)	O1—Cd1—O4—N7	88.4 (5)

## References

[bb1] Bessel, C. A., See, R. F., Jameson, D. L., Churchill, M. R. & Takeuchi, K. J. (1993). *J. Chem. Soc. Dalton Trans.* pp. 1563–1576.

[bb2] Bruker (1997). *SMART* and *SAINT* Bruker AXS Inc., Madison, Wisconsin, USA.

[bb3] Sheldrick, G. M. (1996). *SADABS* University of Göttingen, Germany.

[bb4] Sheldrick, G. M. (2008). *Acta Cryst.* A**64**, 112–122. 10.1107/S0108767307043930 18156677

[bb5] Yang, Z. N. & Sun, T. T. (2008). *Acta Cryst.* E**64**, m1374. 10.1107/S1600536808031152 PMC2959550 21580830

